# Interdisciplinary network care collaboration in Parkinson’s disease: a baseline evaluation in Germany

**DOI:** 10.1186/s42466-023-00300-5

**Published:** 2024-01-11

**Authors:** Carina Lummer, Carsten Eggers, Andreas Becker, Fenja Demandt, Tobias Warnecke, Parkinson Netzwerke Deutschland e.V.

**Affiliations:** 1 OptiMedis AG, Buchardstraße 17, 20095 Hamburg, Germany; 2 Department of Neurology, Knappschaftskrankenhaus Bottrop GmbH - Academic Teaching Hospital of the University of Duisburg-Essen, Osterfelder Str. 157, 46242 Bottrop, Germany; 3 Department of Neurology, SRH-Kurpfalzkrankenhaus Heidelberg - Academic Teaching Hospital of the University of Heidelberg, Bonhoefferstrasse 5, 69123 Heidelberg, Germany; 4 Institute for Applied Health Services Research, Schiffbauerdamm 12, 10117 Berlin, Germany; 5 Department of Neurology and Neurorehabilitation, Klinikum Osnabrück - Academic Teaching Hospital of the University of Münster, Am Finkenhügel 1, 49076 Osnabrück, Germany

**Keywords:** Parkinson’s disease, Interdisciplinary network, Baseline evaluation, Communication, Care collaboration

## Abstract

**Background:**

The strengthening of interdisciplinary care collaboration in Parkinson's disease is taking on increasing importance in daily medical routine. Therefore, care providers worldwide are organizing themselves in disease-specific regional network structures. However, the existing networks are heterogeneous, and the driving key players are yet unidentified.

**Objectives:**

To systematically identify key factors of the composition of health care professionals, who are initially interested in the development of a Parkinson network for interdisciplinary care collaboration, their motivation, and expectations, we conducted a basic evaluation in three different German regions covering a total number of 23,405 people with Parkinson’s.

**Methods:**

A specially developed semi-open questionnaire focusing on socio-demographic information, ways of contact, interdisciplinary collaboration, and connectedness was used. Statistical analyses were performed based on a predesigned codebook.

**Results:**

The most crucial professions were outpatient therapists (physio-, occupational-, speech therapists) (36.7%), average case load of 10.1 patients/3 months and inpatient movement disorder specialists (21.1%), average case load of 197.4 patients/3 months. Before implementation of PD networks, 48.9% of outpatient therapists did not have any contact with neurologists. 58.9% of caregivers considered the current frequency of collaboration to be insufficient. The lack of political support as well as a lack of time were identified as main hurdles to increased collaboration.

**Conclusion:**

The identified driving forces in strengthened care collaboration are assigned to different healthcare sectors. This makes networks which provide tools for specialized education and interdisciplinary, cross-sectoral communication indispensable. For an areawide rollout, a rethinking of political frameworks towards network care is strongly necessary.

## Introduction

Parkinson’s disease (PD) is the second most common neurodegenerative disease [1]. PD is incurable and manifests in motor as well as non-motor symptoms which are individually highly heterogenous [2]. The therapeutic options are very complex due to the variability of symptom combinations and require the regular involvement of specialists and numerous outpatient and inpatient health care professionals (HCP) [3]. In view of the chronification of PD, there are profound life events of the affected persons and their relatives due to a multitude of behavioral and psychological impairments. For appropriate care and optimal support, an interdisciplinary care approach is necessary, considering individual needs as well as the complexity of the disease. Worldwide, the realization of such an approach is achieved by the implementation of multidisciplinary PD networks. Such networks are characterized by an association of all individual actors and institutions from the corresponding region involved in the provision of care. International evidence shows that the work in multidisciplinary networks has a positive effect on care which is established in the reduction of mortality, the increase of life quality and patient satisfaction, better access to specific therapy as well as the reduction of health care costs [4–6].

In accordance with this international development, different regions in Germany have implemented interdisciplinary PD networks throughout the last years. As of today, there are 15 regional PD networks in Germany. Members and partners of the networks are all care providers associated with PD, as well as support groups and caregivers. The networks focus on intensified outpatient care and general local hospitals supported by PD specialized in- and outpatient centers [7].

The regional networks in Germany organize themselves on their own responsibility but are supported by the umbrella institution Parkinson Netzwerke Deutschland e.V. (PND e.V.), the German PD Network Association. PND e.V. defines a network as a “interdisciplinary networked, dynamic, agile, and self-learning structure that aims to improve healthcare delivery mostly indication based. This goal is to be achieved by promoting knowledge, communication as well as coordination.” Regionally, the networks are developing and implementing various measures based on the respective care challenges to achieve the three defined sub-goals. A core element offered homogeneously across all networks is the organization of regular interprofessional network meetings. The network meeting is usually also the starting point for the implementation of a new regional network.

To gain a better understanding of the composition of HCPs, who are initially interested in the development of a PD network as well as their motivation, concerns, and expectations, we conducted a baseline analysis of the driving forces in three newly established networks in different regions of Germany. Such a baseline exploration is a key component for the successful and consistent implementation of PD networks [8] but has not been performed systematically in the past.

## Methods

### Study region

**Fig. 1:**
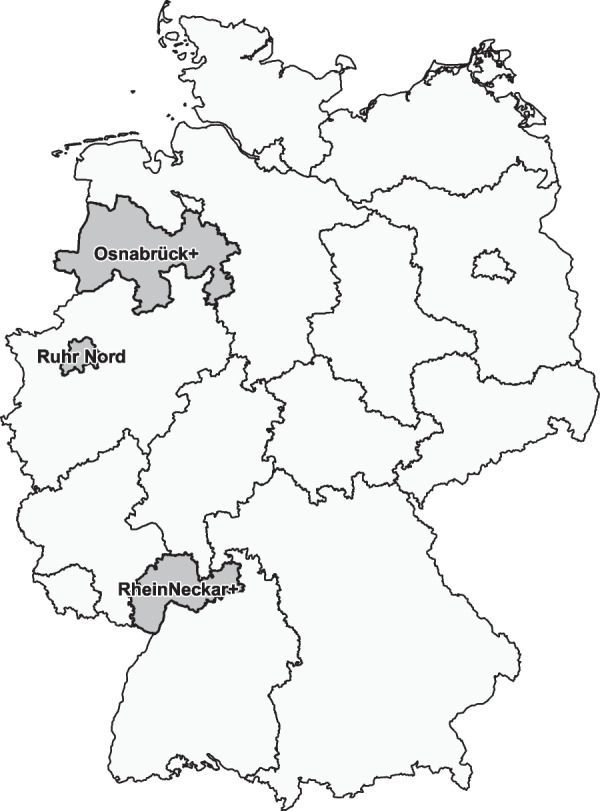
Geographical location of the surveyed networks within Germany

For the conduction of the study, we chose three regions in Germany which within the year 2022 newly implemented interdisciplinary networks. Those were the regions of the PD network Osnabrück+ (PNO+), the PD network Ruhr Nord (PNRN) as well as the PD network RheinNeckar+ (PNRN+). The geographical location within Germany is shown in Fig. 1.

For quality assurance and following recommendations from the Dutch ParkinsonNet [8], all networks were initiated by PD experts who are renown and connected in the scene. All three regions differ from one another as they do have different care structures and HCP densities. The predefined network regions consist of different types of cities and municipalities and therefore have different levels of differentiated infrastructure. The PNRN+ is the largest predefined network region and consists of 15 cities and counties including three metropolitan cities (> 100.000 inhabitants). The total population of the region of the PNRN+ is approximately 2.42 million inhabitants. The predefined region of the PNO+ includes nine cities and counties, of which one is metropolitan. The total population of the region of the PNO+ is approximately 1.64 million inhabitants. The region of the PNRN consists of four cities or counties and has a population of approximately 0.67 million inhabitants. Three of the included cities count as metropolitan. In total, the three considered regions have a population of approximately 4.73 million inhabitants. Among them, approximability 23,405 are suffering from PD. The overall number breaks down into appr. 8900 PwP in the region of the PNO+ , appr. 3340 PwP in the region of the PNRN and appr. 11,165 PwP in the region of the PNRN+ [9].

The survey was conducted at the first interdisciplinary meeting of each network. The invitation to the first meeting functioned via open call for participation to all the relevant HCPs in the predefined regions based on internet research. HCPs were then invited following PND e.V. standards for invitation—with a postal invitation and—where available—via email. HCPs of the following professions including outpatient as well as inpatient sector were invited: neurologists, speech therapists, occupational therapists and physiotherapists, psychotherapists, art therapists, musical therapists, ophthalmologists, pharmacists, geriatricians, (neuro-)psychologists, general practitioners, nursing facilities, podiatrists, medical supply stores, urologists, dentists, rehabilitation physicians, self-help groups and public health departments [5]. There was no knowledge regarding the grade of PD specialization of the HCPs, thus all predefined caregivers of the named professions in the region were invited. In the PNO+, initially 1,015 HCPs were contacted, in the PNRN, 420 HCPs were contacted and the PNRN+ reached out to 3,312 HCPs.

### Questionnaire development and procedures

The questionnaire was developed based on the goals of the network implementation and the experiences of the social network analysis carried out in the Parkinson network Münsterland+ (PNM+), which was the first one of its kind in Germany [10]. We used a semi-open questionnaire, consisting of questions with response scales, questions with single and multiple response options as well as (supplementary) free-text-fields.

The questionnaire was divided into four categories:

1. Socio-demographic information: Regarding sociodemographic data, we intended to evaluate which professions the participants belonged to. We also asked for postal codes for the purpose of the regional origin. In addition, we assessed the number of PwP from each caregiver per quarter.
2. Ways of contact and communication: In the second part of the questionnaire, the goal was to evaluate the current frequency of communication between the caregivers as well as the current methods of communication. For the former purpose, we asked participants to rank the frequency of contact with their own and other professional groups during their work week. For the second purpose, we asked for the most frequent used ways of contact.
3. Interdisciplinary collaboration: We asked the participants for their level of satisfaction with the frequency of collaboration with their own as well as other disciplines. Also, we asked the participants for their motivation to intensify (inter)disciplinary exchange. We also evaluated the participant’s thoughts about existing hurdles as well as success factors in the current collaboration over free-text-responses.
4. Connectedness: In the last part of the questionnaire, we evaluated the current level of (inter-)disciplinary connectedness. We therefore determined the satisfaction level with different predefined sectors of collaboration. We then asked participants for their hoped-for effects of a PD network and their partnership in it.

The survey was carried out on a paper-and-pencil basis. Everyone at the network meeting was invited to participate in the anonymous survey. To reduce biases due to information received from the network founders in the first meeting as well as due to interconnections throughout the meeting, the survey was filled out by the participants before the meeting started.

### Statistical analysis

Data were analyzed using SPSS (Statistical Package for the Social Sciences), version 27.0. For the analysis a predeveloped codebook was used, which was identical for all three networks. Questionnaires with incomplete data were excluded in the regarded parts of the analysis. The analysis was carried out in predefined categories (response scales, single response options, multiple response options). Free-text-responses were categorized based on similar contents of statement. Ordinal variables regarding the frequency of interdisciplinary communication were calculated using the mean value of the in single response scales described frequency per subgroup over all networks. Variables concerning the level of motivation were analyzed using descriptive statistics based on single response rating scales. Variables regarding the level of satisfaction with the current connectiveness were calculated using the mean value of the in single response scales described satisfaction per question over all networks. Values were described in percentage shares.

## Results

### Socio-demographic information

#### Professional composition of participants

Over all three networks, a total of 128 participants took part in the survey. 60 participants were from the region of the PNO (5.9% of the originally contacted HCPs)+, 32 from the PNRN region (7.6% of the originally contacted HCPs) and 36 from the RNRN+ (1.1% of the originally contacted HCPs) region. The disciplinary composition of participants is shown in Table 1.

**Table 1:** Composition of disciplines at first network meeting

Discipline	PNO+	PNRN	PNRN+	All networks
Neurologist (inpatient)	13	7	7	27
Neurologists (outpatient)	4	2	2	8
Therapy (inpatient)	8	4	8	20^1^
Therapy (outpatient)	20	12	15	47^2^
Parkinson’s Nurse	3	1	–	4
Others^3^	12	6	4	22
Sum	60	32	36	128

^1^Speech therapists (9), Physiotherapists (5), Occupational therapists (2), Subgroup not specified (4)

^2^Speech therapists (18), Physiotherapists (9), Occupational therapists (14), Subgroup not specified (6)

^3^Neuropsychology (3), PwP (5), Representative public health sector (1), Medical supply store (6), Psychotherapy (1), Neurosurgery (2), Consultant (1), Professional group not assignable (3)

#### Regions of origins

In the PNO+, 61.7% of the participants came from the county of Osnabrück. 43.2% of those participants lived in the city directly, the other 56.8% in surrounding semi-rural areas close to the city. The rest of the participants came from rural areas (38.3%). The greatest distance from the participants home region to the meeting location was 160 km. In the PNRN, 60.1% of the participants came directly from Bottrop where the meeting took place. The rest of the participants arrived from neighboring cities (23.1%) and rural areas (16.8%). The greatest distance from the participants home region to the meeting location was 53 km. In the PNRN+, 54.8% came directly from Heidelberg where the meeting took place. The rest of the participants lived in neighboring cities (3.2%) and surrounding rural areas (42%). The greatest distance from the participants home region to the meeting location was 62 km.

#### PwP treated per quarter

Across all networks, neurologists in the clinic reported of a treatment average of 197.4 PwP per three months (mean value over all three networks). Outpatient neurologists across all networks treated an average of 80 PwP per three months. Therapists in the inpatient sector treated 19.3 PwP per three months. Therapists in the outpatient sector treated 10.1 PwP per three months. Those divided in 5.8 PwP for occupational therapists, 10.3 for speech therapists and 11.6 PwP for physiotherapists (6 therapists did not specify their subgroup i.e., physiotherapist, occupational therapist, speech therapist).

### Ways of contact and communication

#### Current frequency of communication

The average frequency of disciplinary communication between therapists within their own professional group differed between the outpatient and the inpatient sector. The communication in the outpatient sector is shown in Table 2 (e.g., 44.4% of the physiotherapists reported to not communicate with occupational therapists at all).

**Table 2:** Frequency of interdisciplinary communication between therapeutic subgroups

Discipline communicated with		Physiotherapists	Occupational therapists	Speech therapists
Discipline answering*				
*Physiotherapists*				
No communication at all		0.222	0.444	0.444
Communication on 2–3 days of the week		0.111	0.333	0.111
Communication on 4–5 days of the week		0.444	–	0.222
*Occupational therapists*				
No communication at all		0.389	0.143	0.357
Communication on 2–3 days of the week		0.333	0.143	0.428
Communication on 4–5 days of the week		0.056	0.571	–
*Speech therapists*				
No communication at all		0.167	0.278	0.389
Communication on 2–3 days of the week		0.111	0.278	0.333
Communication on 4–5 days of the week		0.389	0.167	0.056

*Not all therapists did give an answer

The disciplinary communication between neurologists in the outpatient setting within their own profession was described as followed: 25% did not communicate with other neurologists at all, 12.5% communicated on 2–3 days of the work week and 62.5% were in contact with other neurologists on 4–5 days of the work week.

The current frequency of interdisciplinary communication between therapists and neurologists in outpatient setting was reported as follows: 48.9% of therapists did not communicate with neurologists at all, 40.0% were communicating on 2–3 days of the work week and 4.26% nearly on 4–5 days of the work week. 12.7% of the outpatient therapists surveyed did not leave an answer.

#### Ways of communication

As most important way of communicating was named the phone (82%), followed by e-mail (57.8%), fax (13.3%), physical personal exchange (10.2%), reports (3.1%) as well as digital tools (2.3%). 60% of the neurologists and 74.6% of the therapists reported using the phone, 17.1% of the neurologists and 41.8% of the therapists reported using E-Mail and 8.6% of the neurologists and 4.5% of the therapists communicated via Fax.

### Interdisciplinary collaboration

#### Current frequency of collaboration

**Fig. 2:**
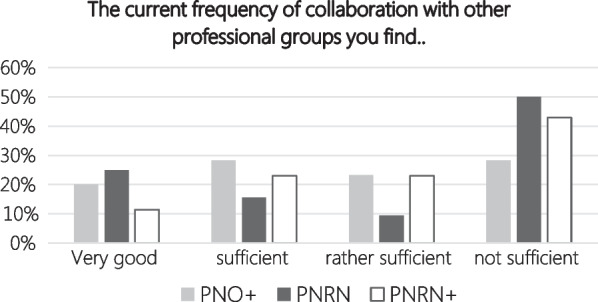
Satisfaction with current frequency of collaboration

Over all three networks (mean value) 18.8% of participants experienced the frequency of collaboration with other professional groups as very good, 22.3% as sufficient, 18.5% as rather sufficient and 40.4% as not sufficient. The surveyed satisfaction of frequency of collaboration in each network is shown in Fig. 2.

#### Motivation for the intensification of exchange

Concerning the motivation for intensification of exchange within the own disciplines we reached the in Table 3 shown answers.

**Table 3:** Motivation for intensification of disciplinary exchange

Network	PNO+	PNRN	PNRN+	All networks (mean value)
*Level of motivation*				
Very motivated	0.84	0.69	0.58	0.703
Rather motivated	0.15	0.22	0.33	0.233
Rather not motivated	0.01	0.09	0.03	0.043
Not motivated	–	–	0.06	0.02

Concerning the motivation for intensification of exchange with other disciplines we reached the in Table 4 shown answers.

**Table 4:** Motivation for intensification for interdisciplinary exchange

Network	PNO+	PNRN	PNRN+	All networks (mean value)
*Level of motivation*				
Very motivated	0.71	0.69	0.69	0.697
Rather motivated	0.29	0.28	0.31	0.293
Rather not motivated	–	0.03	–	0.01
Not motivated	–	–	–	–

#### Hurdles and success factors in current collaboration

The lack of time was identified as the most relevant barrier (42%) followed by a lack of structures for collaboration (23%), limited reachability of the partners (13%), uncertainty of the correct contact partner for collaboration (12%) and lack of financial compensation (10%). Success factors were coordinated treatment approaches (22%), increasing exchange and knowledge transfer (19%), gain of information (19%), transparency in the work of each discipline (13%) as well as existing network groups (13%). Other success factors were named sporadically (14%).

### Connectedness

#### Satisfaction with the current connectedness

The results reached for the satisfaction with the current connectedness are shown in Table 5.

**Table 5:** Level of satisfaction with current connectedness

Satisfaction…	Very satisfied	Satisfied	Rather unsatisfied	Very unsatisfied
…with the current collaboration with other professions	0.104	0.456	0.374	0.066
…with the current collaboration with your own professions	0.183	0.481	0.252	0.084
…with the current offers for interconnection in the region	0.054	0.244	0.569	0.133
…with political engagement for integrated care in the region	0.017	0.164	0.498	0.321

#### Hoped-by effects from the network implementation

Participants hoped for an increase of quality of PD care structures (84.7%), better collaboration with network partners in the treatment of PD (80.7%), a regular exchange regarding the current care situation with other network partners (72%), an increase of PD specific knowledge (65.3%) as well as an equal participation in care of all care providers (61.7%) (multiple answers possible). Neurologists especially wished for a strengthened collaboration with physiotherapy, occupational therapy and speech therapy, other neurologists and with Parkinson Nurses. Therapists on their behalf hoped for a strengthened collaboration with neurologists and general practitioners, within their own discipline as well as with nurses.

## Discussion

Within the landscape of German PD networks, this is the first study evaluating basic key factors of the composition of professionals, who are initially interested in the development of a network as well as their motivation, concerns, and expectations towards the regional implementation of network structures. Three newly founded networks were surveyed, together covering a population of 4.73 million inhabitants of which an estimated number of 23,405 people is suffering from PD. The most strongly represented professions throughout all three regions at the founding meetings were therapists from the outpatient sector and movement disorder specialists from the clinic. As it is known to be of great importance to early identify key people driving interdisciplinary care collaboration forward [5, 8], those groups were identified for taking on that role in the German networks.

The results of the sociodemographic analyses show that therapists from the outpatient sector who want to be involved in the network treat an average of 10.1 PwP per three months. As opposed to this number, outpatient neurologists who are particularly involved in PD care are treating an average 80 PwP per three months. Inpatient neurologists have a high degree of specialization, with an average treatment rate of approximately 200 PwP per three months. Data from the Netherlands [6] having an equal PD prevalence as Germany, with sophisticated specialist trainers showed an increase in the case load of 35% (physiotherapists), 42% (speech-language therapists), 65% (occupational therapists) over a period of five years. In Germany, regions without a PD network do not offer specialist PD education for therapists. However, regarding the current case load in the surveyed region where no specialized PD education was available, there already is a comparable case load among the therapists intrinsically motivated. It can be assumed, that after the implementation of education program analog to the Netherlands, it will be possible to achieve a significant increase in patient numbers and thus also in PD specific expertise [4] in the next few years. In this way, an increase in the number of PwP treated quarterly per therapist may be expected to an average of 14 PwP within five years.

Regarding the analysis of communication and ways of contact, we found that the most common communication channels by far are telephone and email, while digital tools do not yet play a relevant role. The communication of the therapists in the outpatient setting mainly takes place among their own disciplinary subgroup, but not between the groups. There is hardly any communication between therapists and neurologists in the outpatient area. Hence, a timely implementation of facilitated communication structures in terms of online tools to which the relevant players have access is a core task for the German networks in order to strengthen care collaboration. With the aim of bringing interprofessional communication into the reimbursement structure of the statutory health insurance funds, this should be a uniform and according to the high data privacy regulations suitable structure throughout Germany. Here, the now digitized, guideline-based quickcards from the PNM+ might serve as a relevant and scalable tool [11, 12]. To the best of our knowledge, in networks worldwide there is no structured, guideline-based and areawide accessible digital tool for the validated communication and systematic feedback between the main care providers for PD [13]. The digital quickcard approach via online platform therefore cannot only contribute to improved connectedness nationwide, but also internationally and cross-disease. Also, the telematic infrastructure with its various applications (messenger, electronic prescription, electronic health record), might be able to support this.

The results of the analysis of interdisciplinary communication show that half of the caregivers consider the frequency of current collaboration to be insufficient, although there is a very high motivation to exchange information with all other disciplines. Accordingly, a crucial activity should be to increase eye-to-eye communication and to communicate the possibilities of communication to network partners. For this purpose, the topic of communication with partners should be included as a separate module in the further training curriculum of the networks. International experience can serve as a role model here [14–16], but the material must be adapted to the German structures and needs of the HCPs. Also, as described above, the implementation of tools for an improved communication and feedback structure is desired and necessary.

Concerning the connectedness, a lack of offerings and a lack of political support were identified as a driving force for participation. There is a high intrinsic motivation between the caregivers to network across all professional groups and to establish corresponding structures. This shows that, in addition to the intrinsic motivation of the participants and the recognition of the need for regional cooperation, the political framework conditions for such activities are required. Compared to other countries [8], in Germany, with a multi-player health system of 96 statuary health insurances [17] and more than 40 private health insurance, the negotiations for an establishment of reimbursed networks, which could contribute to savings within the health care system, are quite challenging. PND e.V. aims to increasingly advocate for the establishment and further development of network structures in the political environment. Ultimately, these regional networks should demonstrate an improvement of population health and / or reduce healthcare-associated costs, which would require a further analysis.

Some limitations of the study must be named: By initiating the network meeting via open call for participation, it can be assumed that there is a natural selection of motivated providers who will then also come to the meeting. The results therefore cannot be considered as a representation of the overall experience in the region. In process of further developing the networks, there are plans for the conduction of in-depth analysis of the care situation of individual regions before network implementation. Also, for a wider roll-out strategy of the network, described measures like the open call for invitation might not be enough, especially regarding small, specialized groups, e.g., neuro-urologists, who tend not to identify themselves as specialists for PD. [2]. For these groups, discipline-specific calls for participation are needed. However, via PND e.V., we now have a greater reachability and a chance of merging those regional experts into a powerful group.

## Conclusions

The Netherlands was the first country to implement, reimburse and centralized coordinate the treatment of PwP in network structures and can therefore function as a role model. In 2014, ParkinsonNet tried to transfer the ParkinsonNet model on a Nordrhein region in Germany and failed within the process [8]. Transferability studies show that although the structural idea of ParkinsonNet could be transferred also at that time (e.g., through translation of education material), the key people needed to set up and continue the network were missing. Through the development, experiences, and learning processes of the past five years as well as the professionalization of the networks via the PND e.V., we are now able to identify, train and promote those key people. By the structural support of intrinsic motivated people, we can obtain that the process not only starts, but can succeed in the long term.

Furthermore, ParkinsonNet identified one further main factor in the transferability of a network which is the political context. Here, the key thought is whether PD is a priority disease for decision makers and funders and whether centralized integrated care is in the focus of health policy development. Regarding the first point, PD with appr. 400,000 affected people might not be regarded as highly prioritized at first. However, PD is associated with a high number of co-morbidities as well with a long journey through the healthcare system with numerous cases of misdiagnosis which prolong the expensive process of diagnosis. Also, PD is internationally proven as a model disease for the implementation of innovative care models [1]. Therefore, there is a high transferability of PD structures in other neurodegenerative diseases, like dementia. Concerning the question of integrated care focuses, development in Germany politics shows an increased focus on regional low-threshold care. This is where PD networks with a very well-developed, nationwide known structure, can also be synchronized as an indication specific to other regional developments, such as health kiosks. For a nation-wide roll-out of structures of the network idea, irrespective of the indication, a rethinking of the reimbursement structures of caregivers towards shared savings is provided.

## Data Availability

The datasets during and/or analysed during the current study available from the corresponding author on reasonable request.

## Abbreviations

HCP: Healthcare professional
PD: Parkinson’s disease
PND e.V.: German Parkinson Network Association (Parkinson Netzwerke Deutschland e.V.)
PNM+: Parkinson Network Münsterland
PNRN: Parkinson Network Ruhr Nord
PNRN+: Parkinson Network Rhein Neckar+
PNO+: Parkinson Network Osnabrück+
PwP: People with Parkinson’s
